# Optical coherence tomography and fractional flow reserve guided treatment of woven coronary artery anomaly presenting as acute myocardial infarction: A case report

**DOI:** 10.1097/MD.0000000000019163

**Published:** 2020-02-14

**Authors:** Fangfang Wang, Jiangli Han, Lijun Guo

**Affiliations:** a Department of Cardiology and Institute of Vascular Medicine, Peking University Third Hospital; b NHC Key Laboratory of Cardiovascular Molecular Biology and Regulatory Peptides; c Key Laboratory of Molecular Cardiovascular Science, Ministry of Education; d Beijing Key Laboratory of Cardiovascular Receptors Research, Beijing, China.

**Keywords:** acute myocardial infarction, fractional flow reserve, optical coherence tomography, woven coronary artery

## Abstract

**Rationale:**

Woven coronary artery is a very rare congenital anomaly which may lead to acute coronary syndrome in previous literatures. At present, there is no consensus on the treatment of this coronary artery abnormality.

**Patient concerns:**

A 48-year-old male was admitted to the other hospital because of persistent chest pain. The electrocardiogram showed an ST-segment elevation in the v1-v5 lead and the patient was diagnosed with acute anterior myocardial infarction. Coronary angiography revealed 2 lumens in the proximal segment of the left anterior descending artery. Then the patient was transferred to our hospital for further diagnosis and treatment.

**Diagnoses:**

The patient was diagnosed with acute myocardial infarction and woven coronary. Transthoracic echocardiography showed left ventricular anterior wall segmental motor abnormalities.

**Interventions:**

Optical coherence tomography (OCT) and fractional flow reserve (FFR) guided percutaneous coronary intervention was performed successfully.

**Outcomes:**

During the follow-up period of 4 years, the patient remained asymptomatic and no adverse events.

**Lessons:**

Although the significance of blood flow limitation in one of the lumens detected by FFR is unclear, this strategy of OCT and FFR-guided treatment in woven coronary artery combined with acute coronary events still shows its feasibility.

## 1 Introduction

Woven coronary artery (WCA) is often considered a rare congenital vascular malformation. The typical pathological feature is that the lumen of 1 or more segments of the coronary artery is separated into small passages by small blood vessels that are intertwined. These vascular networks are recombined into the normal lumen at the distal end of the lesion. Such malformations may occur in any segment of the coronary arteries, and relatively few simultaneous occurrences in multiple blood vessels. Although WCA is generally considered to be benign, there are reports of related ischemia, thrombosis, atherosclerotic plaque formation.^[1–3]^ Optical coherence tomography (OCT) is critical for diagnostic and differential diagnosis, especially for intracoronary thrombosis, spontaneous coronary dissection, or chronic complete obstruction with bridging collaterals. The treatment strategy of WCA patients depends on the clinical situation of the specific case, and there are no relevant guidelines or consensus.

## 2 Case report

**Figure 1:**
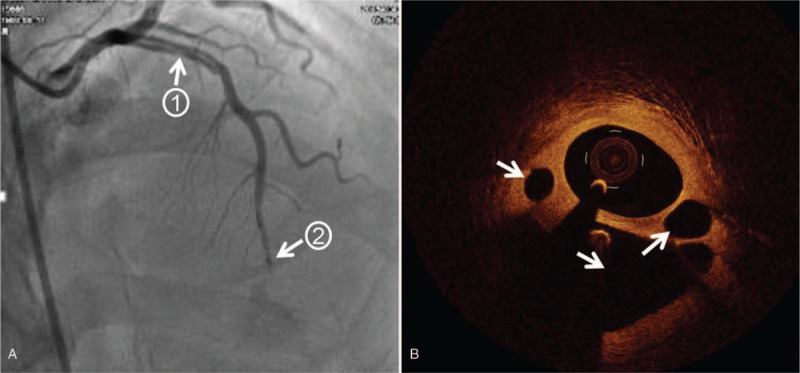
Images before intervention therapy. A, Coronary angiography showing a double-chamber blood flow in the proximal LAD artery (arrow 1) and slightly slow blood flow in the distal (arrow 2). B, OCT showing cavities of different sizes in the proximal segment of the LAD artery (arrow 3), and no obvious atherosclerotic plaques, thrombosis, or dissection. LAD = left anterior descending, OCT = optical coherence tomography.

**Figure 2:**
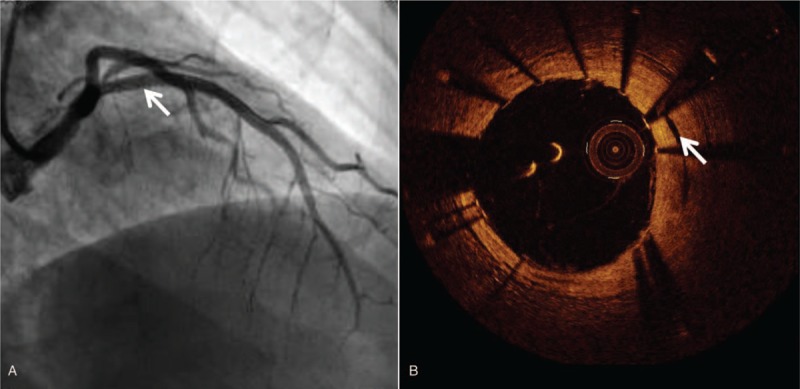
Images after intervention therapy. A, PCI was performed with a 3.0/33 mm DES implanted in the proximal LAD artery (arrow) and there were no interventional complications. B, OCT re-examination finding that the intertwined small blood vessels around the main lumen in the proximal LAD artery (shown as cavities before) were almost occluded (arrow). PCI = percutaneous coronary intervention, DES = drug-eluted stent, LAD = left anterior descending, OCT = optical coherence tomography.

A 48-year-old male was admitted to the other hospital because of persistent chest pain. The electrocardiogram showed an ST-segment elevation in the V1-V5 lead and the patient was diagnosed with acute anterior myocardial infarction. Coronary angiography revealed at least 2 lumens in the proximal segment of the left anterior descending (LAD) artery. Then the doctor transferred the patient to our hospital 1 week later for further diagnosis and treatment. Echocardiography in our hospital showed akinesia at anterior walls, and ejection fraction was 54%. Coronary angiography showed a double-chamber blood flow in the proximal LAD artery, and the distal blood flow was slightly slow (Fig. 1A and Supplementary material online, Video S1). Further OCT examination showed that there were cavities of different sizes in the proximal segment of the LAD artery, and there were no obvious atherosclerotic plaques and thrombosis (Fig. 1B and Supplementary material online, Video S2). The diagnosis was considered to be a WCA. Because there is currently no treatment recommendation for this coronary anomaly, we decided to perform fractional flow reserve (FFR) to determine if the intervention was needed. Subsequent FFR results were 0.72 and percutaneous coronary intervention (PCI) was performed with a 3.0/33 mm drug-eluted stent implanted in the proximal LAD artery. FFR final result was 0.86 and the patient did not have any interventional complications (Fig. 2A and Supplementary material online, Video S3). OCT re-examination revealed that the intertwined small blood vessels around the main lumen (shown as cavities before) were almost occluded (Fig. 2B and Supplementary material online, Video S4). During the follow-up period of 4 years, the patient remained asymptomatic and no adverse events.

## 3 Discussion

Since 1988, there have been dozens of WCA case reports in PubMed, the vast majority of cases were accidentally discovered in coronary angiography. In the reported cases, the minimum age was 9 months, so some speculated that this was a congenital anatomic abnormality.^[4]^ As a rare coronary anatomical abnormality, the etiology of WCA is still unclear, and clinical processes are also lacking in specificity. Suzuki et al^[5]^ performed a tunnel biopsy on patients with WCA who were prompted by coronary angiography. Histopathological results showed that smooth muscle cells were seen between the bilateral endothelial layers and no atherosclerotic plaques were seen. Combined with the patient's background, the authors speculate that the formation of the braided structure is likely to be recanalized due to vasospasm, rather than congenital vascular abnormalities, or recanalization of thromboembolism or healing of plaque rupture. This also suggests that there is a certain limitation in the diagnosis of WCA by coronary angiography alone.

Intravascular ultrasound, OCT, and coronary endoscopy can help to determine the lumen and wall structure of different tunnels and to make a clear diagnosis. OCT utilizes light-based technology to obtain 360° cross-sectional images of a coronary artery with a continuous pullback image of an arterial segment. In WCA patients, OCT shows multiple spiral tunnels in the local lumen, separated by fibrous tissue with high signal intensity and low signal attenuation. And each tunnel should have a relatively complete 3-layer vascular structure, which is to identify whether the thrombus is recanalized. The improved resolution allows accurate identification of the 3 layers of the normal arterial wall, delineation of plaque morphology which sheds lights on diagnosis and management of WCA.^[6,7]^ In the absence of coronary atherosclerotic plaque, stenosis, or thrombosis, blood flow in WCA patients is often unrestricted, so it is considered to be a benign variant. However, there were also WCA patients presenting with acute coronary syndromes or even sudden death.^[8,9]^ Therefore, the clinical manifestations of structural abnormalities in the WCA are complex. Sometimes, interventional cardiologists choose more aggressive strategies, such as PCI or coronary artery bypass grafting.^[10]^

The clinical manifestations of the case we reported were acute myocardial infarction. Coronary angiography showed that the blood flow of the left LAD branch of the braided coronary artery was slowed down, so there was a possibility of limited blood flow. OCT showed multiple spiral tunnels in the local lumen and each tunnel should have a relatively complete 3-layer vascular structure, WCA was diagnosed. FFR is a well-established pressure-wire–based technique that is used to assess the functional severity of coronary lesions and FFR≤0.75 always defined as coronary arteries with flow-limiting lesions and PCI was recommended.^[11–13]^ The FFR results showed less than 0.75, so we determined to do PCI. The success of PCI and good follow-up results indicate that PCI is an alternative treatment. Our experience provides a new assessment tool and treatment strategy for WCA combined with acute coronary events patients.

## 4 Conclusion

WCA may cause myocardial ischemia even myocardial infarction. PCI may be one of the treatment options for this anomaly. Although the significance of blood flow limitation in one of lumens detected by FFR is unclear, this strategy of OCT and FFR-guided treatment in WCA combined with acute coronary events still shows its feasibility.

## Author contributions

**Supervision:** Lijun Guo.

**Writing – original draft:** Fangfang Wang.

**Writing – review & editing:** Jiangli Han.
